# Numerical study of non-Darcy hybrid nanofluid flow with the effect of heat source and hall current over a slender extending sheet

**DOI:** 10.1038/s41598-022-20583-z

**Published:** 2022-09-29

**Authors:** Zehba Raizah, Hussam Alrabaiah, Muhammad Bilal, Prem Junsawang, Ahmed M. Galal

**Affiliations:** 1 Department of Mathematics, College of Science, King Khalid University, Abha, Saudi Arabia; 2 College of Engineering, Al Ain University, Al Ain, United Arab Emirates; 3 Mathematics Department, Tafila Technical University, Tafila, Jordan; 4 Department of Mathematics, City University of Science and IT, Peshawar, 25000 Pakistan; 5 Department of Statistics, Faculty of Science, Khon Kaen University, Khon Kaen, 40002 Thailand; 6 Department of Mechanical Engineering, College of Engineering in Wadi Alddawasir, Prince Sattam Bin Abdulaziz University, Wadi Alddawasir, Saudi Arabia; 7 Production Engineering and Mechanical Design Department, Faculty of Engineering, Mansoura University, Mansoura, 35516 Egypt

**Keywords:** Engineering, Mathematics and computing

## Abstract

The current evaluation described the flow features of Darcy Forchhemier hybrid nanoliquid across a slender permeable stretching surface. The consequences of magnetic fields, second order exothermic reaction, Hall current and heat absorption and generation are all accounted to the fluid flow. In the working fluid, silicon dioxide (SiO_2_) and titanium dioxide (TiO_2_) nano particulates are dispersed to prepare the hybrid nanoliquid. TiO_2_ and SiO_2_ NPs are used for around 100 years in a vast number of diverse products. The modeled has been designed as a nonlinear set of PDEs, Which are degraded to the dimensionless system of ODEs by using the similarity transformation. The reduced set of nonlinear ODEs has been numerically estimated through bvp4c package. The outcomes are tested for validity and consistency purpose with the published report and the ND solve technique. It has been noted that the energy curve lessens with the influence of thermodiffusion, Brownian motion and rising number of nanoparticles, while boosts with the result of magnetic field. Furthermore, the concentration outline of hybrid nanoliquid improves with the upshot of chemical reaction.

## Introduction

The analysis of fluid flow over a slendering surface has frequent implementations in various fields, containing manufacture of glass, aerodynamic, polymer industry, firmness of plastic slips and metal tubular^1–3^. Gul et al.^4^ examined and evaluated the proficiency of a hybrid nanofluid along an increasing sheet. It was discovered that the magnetism influence altered the instability of liquid. Bilal et al.^5^ employed the PCM methodology to imitate the movement of nanoliquids through a stretchable material with the effects of suction and injection. The physical and chemical properties of nanofluid flow passing through permeable stretching was documented by Safwa et al.^6^. Moreover, Hussain et al.^7^ reported the energy conversions of MHD nanoliquid flow along an elongating surface. Shuaib et al.^8^ described the ferrofluid flow along with the characteristics of energy conveyance through spinning sheet. Hussain et al.^9^ assessed the energy transport through nanoliquid flow over an extending cylinder. Uddin et al.^10^ analysed the energy transmission through water-based nanoliquid across an expanding surface. Rasool et al.^11^ documented the nanoliquid flow across a contracting surface. Ahmad et al.^12^ assessed nanoliquid fluid across a slender stretching sheet.

Hybrid nanofluid has greater thermal efficiency and mostly utilized in industry for cooling purposes^13^. Hybrid nanofluid work in solar energy, energy transition, air conditioners, generators, the vehicle sector, radioactive systems, electrical coolers, ships, biotechnology and transmitters^14–16^. TiO_2_ and SiO_2_ have non-toxic, non-reactive characteristics and absorb UV rays used for skin cancer, drug delivery, recording devices and solar cells^17^. Traciak et al.^18^ conducted an experimentally assessed the density, optical characteristics and surface tension of SiO_2_-containing nanoliquids based on ethylene glycol. Using the bvp4c software, Bhatti et al.^19^ provided a detailed discussion of SiO2 and carbon nanocrystals over an elastic substrate. Ahmed et al.^20^ scrutinized the nanoliquid flow and energy conveyance through Al_2_O_3_ and TiO_2_ nps based nanoliquid, to augments the thermal efficiency of base solvent, such as thermal diffusivity and heat transport coefficient. Khashi'ie et al.^21,22^ highlighted the comportment of Al_2_O_3_-Cu based hybrid nanoliquid flow and its thermal properties as they were driven by an elongating Riga plate. Alwawi et al.^23^ addressed the impact of magnetism on nanofluid streaming in the scenario of coupled convection across a circular cylinder. The findings show that increasing the coupled convection factor's value improves the Nusselt number, velocity, skin friction and rotational velocity while reducing the thermal contour's trends. Abbasi et al.^24^ comparatively reported the thermal assessment of three distinct sorts of nano particulates, including TiO_2_, SiO_2_ and aluminum oxide through curved sheet. Khashi’ie et al.^25^ used Cu-Al_2_O_3_ hybrid nanoparticles to study the Blasius flow across a rotating plate. De^26^ and Mondal et al.^27^ investigated the combined influence of Soret-Dufour interactions in a nanoliquid flow. Recently, a number of investigators have described on the evaluation of hybrid nanoliquid flow over distinct configuration^28–32^.

Hall current can be detected if the fluid density is small, or the magnetic flux amplitude is strong. In many practical operations that call for an intense electric affect and smaller atomic concentration, hall effects should not be undervalued. Electron transport, where electrons move more quickly than ions, is what results in isotropic conductivity. Ohm's law needs to be revised for the purposes to consider the Hall effect. It has several applications in Hall activators, circuits, pumps, electric inverters, turbines and other equipment, Nanoliquid flow with the upshot of Hall current and magnetic effect has drawn the attention of scientists^33,34^. Using an extended sheet, Khan and Nadeem^35^ examined a spinning Maxwell nanoliquid flow with a magnetism, Hall current and kinetic energy. An asymmetrical reactive nanoliquid flow induced by a magnetization revolving plate and the Hall impact is described by Acharya et al.^36^ along with the flow dynamics and energy variations. They found that the energy transference was improved by 84.61% by nanocomposites. The Hall effect in nanofluid flow has recently been the subject of numerous investigations^37–40^.

The purpose of the current assessment is to study the flow features of Darcy Forchhemier hybrid nanoliquid across a slender permeable stretching surface. The consequences of magnetic fields, second order exothermic reaction, Hall current and heat absorption and generation are all accounted to the fluid flow. In the working fluid, SiO_2_ and TiO_2_ nano particulates are dispersed to prepare the hybrid nanoliquid. The modeled has been designed as a nonlinear set of PDEs. Which are transmute to the dimensionless system of ODEs by using the similarity replacement. The reduced set of nonlinear ODEs has been numerically estimated through bvp4c package.

## Mathematical framework

**Figure 1:**
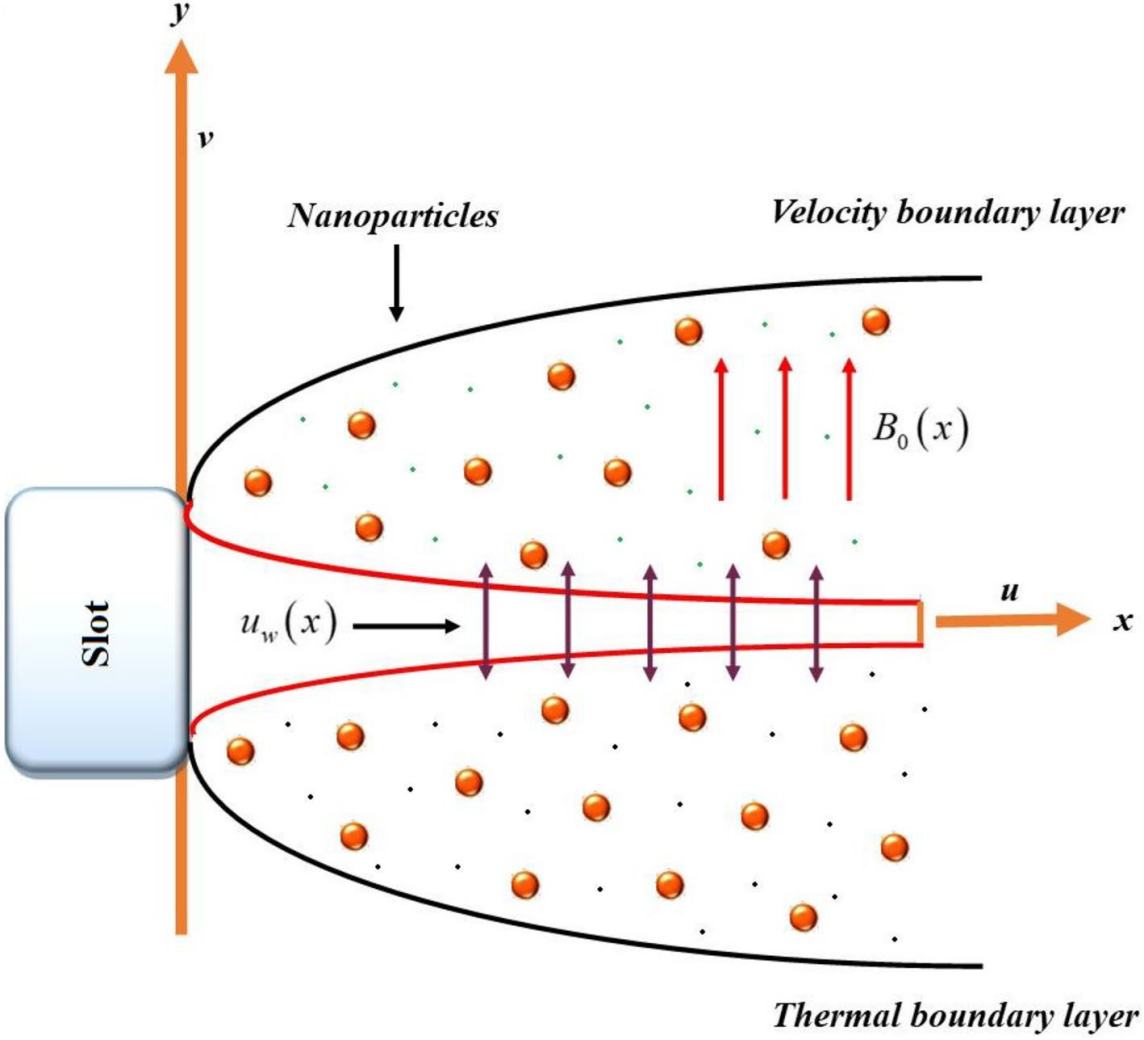
The fluid flow across a slandering expanding cylinder.

We assumed a steady 2D MHD hybrid nanoliquid flow through impermeable slendering substrate. The surface is stretching with velocity $U_{w} \left( x \right) = \left( {x + b} \right)^{n} U_{0} ,$ as described in Fig. 1, where *n* is the power index. The sheet irregularity is assumed as $y = A\left( {x + b} \right)^{{\frac{1 - n}{2}}} ,$ (*A* is the stretching constant). The Hall and magnetic effect are employed for flow motion in *y*-direction. Heat source, Brownian motion, thermo-diffusion and chemical reactions are all observed in current analysis.

The basic equations responsible for the fluid flow are characterized as^41^:

1 $$\frac{\partial u}{{\partial x}} + \frac{\partial v}{{\partial y}} = 0,$$

2 $$\rho_{hnf} \left( {u\frac{\partial u}{{\partial x}} + v\frac{\partial u}{{\partial y}}} \right) = \mu_{hnf} \frac{{\partial^{2} u}}{{\partial y^{2} }} - \frac{{\sigma_{hnf} }}{{1 + m^{2} }}B^{2} \left( x \right)\left( {u + mw} \right) - \frac{{\upsilon_{hnf} }}{{K^{*} }}u - \frac{1}{{\rho_{hnf} }}Fu^{2} ,$$

3 $$\rho_{hnf} \left( {u\frac{\partial w}{{\partial x}} + v\frac{\partial w}{{\partial y}}} \right) = \mu_{hnf} \frac{{\partial^{2} w}}{{\partial y^{2} }} - \frac{{\sigma_{hnf} }}{{1 + m^{2} }}B^{2} \left( x \right)\left( {mu - w} \right) - \frac{{\upsilon_{hnf} }}{{K^{*} }}w - \frac{1}{{\rho_{hnf} }}Fw^{2} ,$$

4 $$\left( {u\frac{\partial T}{{\partial x}} + v\frac{\partial T}{{\partial y}}} \right) = \frac{{k_{hnf} }}{{\left( {\rho C_{p} } \right)_{hnf} }}\left( {\frac{{\partial^{2} T}}{{\partial y^{2} }}} \right) + \left( {D_{B} \frac{\partial T}{{\partial y}}\frac{\partial C}{{\partial y}} + \frac{{D_{T} }}{{T_{\infty } }}\left( {\frac{\partial T}{{\partial y}}} \right)^{2} } \right) + \frac{{Q_{0} \left( {T - T_{\infty } } \right)}}{{\rho C_{p} }},$$

5 $$\left( {u\frac{\partial C}{{\partial x}} + v\frac{\partial C}{{\partial y}}} \right) = D_{B} \left( {\frac{{\partial^{2} C}}{{\partial y^{2} }}} \right) + \frac{{D_{T} }}{{T_{\infty } }}\frac{{\partial^{2} T}}{{\partial y^{2} }} - Kc^{2} \left( {C - C_{\infty } } \right),$$

here, $m = \tau_{e} w_{e}$ is the Hall current, *Kc*^*2*^, *Q*_*0*_, K^*^ and $F = C_{b} /rK^{*1/2}$ are the chemical reaction rate, heat source, permeability factor and non-uniform inertia factor respectively.

The initial and boundary conditions are:

6 $$\left. \begin{aligned} & u = U_{w} \left( x \right) = U_{0} \left( {x + b} \right)^{n} ,\;v = 0,\;w = 0,\;D_{B} \frac{\partial C}{{\partial y}} + \frac{{D_{T} }}{{T_{\infty } }}\frac{\partial T}{{\partial y}} = 0,\;T = T_{w} \;{\text{at}}\;y = A\left( {x + b} \right)^{{\frac{1 - n}{2}}} \\ & u \to 0,\;T \to T_{\infty } ,\;w \to 0,\;C \to C_{\infty } \;as\;y \to \infty . \\ \end{aligned} \right\}$$

The transformation variables are:

7 $$\begin{aligned} & \eta = y\sqrt {\frac{n + 1}{2}\frac{{U_{0} }}{{\nu_{f} }}\left( {x + b} \right)^{m - 1} } ,\;\psi = \sqrt {\frac{2}{n + 1}\nu_{f} U_{0} \left( {x + b} \right)^{m + 1} } \;f\left( \eta \right),\;\varphi \left( \eta \right) = \frac{{C - C_{\infty } }}{{C_{w} - C_{\infty } }}, \\ & w = U_{0} \left( {x + b} \right)^{n} h\left( \eta \right),\;\theta \left( \eta \right) = \frac{{T - T_{\infty } }}{{T_{w} - T_{\infty } }}. \\ \end{aligned}$$

By merging Eq. (7) in Eqs. (1)–(6), we get:

8 $$f^{\prime\prime\prime} + \frac{{\vartheta_{1} }}{{\vartheta_{2} }}\left( {\left( {ff^{\prime\prime} - \frac{2n}{{n + 1}}Frf^{{\prime}{2}} } \right) - \frac{{\vartheta_{3} }}{{\vartheta_{1} }}\left( {\frac{2M}{{\left( {n + 1} \right)\left( {1 + m^{2} } \right)}}} \right)f^{\prime} + \lambda mg} \right) = 0,$$

9 $$g^{\prime\prime} + \frac{{\vartheta_{1} }}{{\vartheta_{2} }}\left( {\left( {fg^{\prime} - \frac{2n}{{n + 1}}Frgf^{\prime}} \right) - \frac{{\vartheta_{3} }}{{\vartheta_{1} }}\left( {\frac{2M}{{\left( {n + 1} \right)\left( {1 + m^{2} } \right)}}} \right)mf^{\prime} - \lambda g} \right) = 0,$$

10 $$\theta^{\prime\prime} + Pr\,\frac{{\vartheta_{4} }}{{\vartheta_{5} }}\left( {f\theta^{\prime} + Nb\,\varphi^{\prime}\theta^{\prime} + Nt\,\theta^{{\prime}{2}} } \right) + Q_{1} \theta = 0,$$

11 $$\varphi^{\prime\prime} + \frac{Nt}{{Nb}}\theta^{\prime\prime} + Lef\varphi^{\prime} - Kr\varphi = 0.$$

here, $$\vartheta_{1} = \frac{{\rho_{hnf} }}{{\rho_{f} }},\,\,\,\vartheta_{2} = \frac{{\mu_{hnf} }}{{\mu_{f} }},\,\,\,\vartheta_{3} = \frac{{\sigma_{hnf} }}{{\sigma_{f} }},\,\,\,\,\vartheta_{4} = \frac{{\left( {\rho C_{p} } \right)_{hnf} }}{{\left( {\rho C_{p} } \right)_{f} }},\,\,\,\,\vartheta_{5} = \frac{{k_{hnf} }}{{k_{f} }}.\,$$

The conditions for system of ODEs are:

12 $$\left. \begin{aligned} & f\left( \eta \right) = \eta \left( {\frac{1 - n}{{1 + n}}} \right),\;Nb\;\varphi^{\prime}\left( \eta \right) + Nt\;\theta^{\prime}\left( \eta \right) = 0,\;f^{\prime}\left( \eta \right) = 1,\;g\left( \eta \right) = 0,\;\theta \left( \eta \right) = 1 \\ & f^{\prime} \to 0,\;g \to 0,\;\theta \to 0,\;\varphi \to 0\;as\;\eta \to \infty \\ \end{aligned} \right\}$$

here, the *M*, $\lambda$, *Pr, Nt, Nb, Fr*, $Q_{1}$, *Le, Gr*, $\delta$,, *Gc* and *Kr* is mathematically expressed as:

13 $$\begin{aligned} & M = \frac{{B_{0}^{2} \sigma_{f} }}{{\rho_{f} T_{\infty } }},\;Pr = \frac{{\mu_{f} \left( {\rho C_{p} } \right)_{f} }}{{\rho_{f} k_{f} }},\;\lambda = \frac{\nu }{{k^{*} b}},\;Nt = \frac{{\tau D_{T} \left( {T_{w} - T_{\infty } } \right)}}{{\nu_{f} T_{\infty } }},\;Fr = \frac{{C_{b} }}{{K^{*1/2} }},\;Nb = \frac{{\tau D_{B} C_{\infty } }}{{\nu_{f} }},\;Q_{1} = \frac{{xQ_{0} }}{{\rho C_{p} }}, \\ & Le = \frac{{\nu_{f} }}{{D_{B} }},\;\delta = A\sqrt {\frac{n + 1}{2}\frac{{U_{0} }}{{\nu_{f} }}} ,\;Gr = \frac{{g\beta_{{T_{f} }} \left( {T_{w} - T_{\infty } } \right)n}}{{U_{w}^{2} }},\;Gc = \frac{{g\beta_{{C_{f} }} \left( {C_{w} - C_{\infty } } \right)n}}{{U_{w}^{2} }},\;Kr = \frac{{Kc^{2} }}{b}. \\ \end{aligned}$$

The physical interest quantities are:

14 $$C_{{f_{x} }} = \frac{{2\tau_{{w_{1} }} }}{{U_{w}^{2} \rho_{f} }},\,\,\,C_{{f_{z} }} = \frac{{\tau_{{w_{2} }} }}{{U_{w}^{2} \rho_{f} }},\,\,\,Nu = \frac{{q_{w} \,\left( {x + b} \right)}}{{\left( {T_{w} - T_{\infty } } \right)k_{f} }},\,\,\,\,Sh = \frac{{j_{w} \,\left( {x + b} \right)}}{{\left( {C_{w} - C_{\infty } } \right)D_{B} }}.$$

where,

15 $$\left. \begin{aligned} & \tau_{{w_{1} }} = \mu_{hnf} \left( {\frac{\partial u}{{\partial y}}} \right)_{{y = A\left( {x + b} \right)^{{\frac{1 - n}{2}}} }} ,\;\tau_{{w_{2} }} = \mu_{hnf} \left( {\frac{\partial v}{{\partial y}}} \right)_{{y = A\left( {x + b} \right)^{{\frac{1 - n}{2}}} }} , \\ & q_{w} = - k_{hnf} \left( {\frac{\partial T}{{\partial y}}} \right)_{{y = A\left( {x + b} \right)^{{\frac{1 - n}{2}}} }} ,\;j_{w} = - D_{B} \left( {\frac{\partial C}{{\partial z}}} \right)_{{y = A\left( {x + b} \right)^{{\frac{1 - n}{2}}} }} . \\ \end{aligned} \right\}$$

The dimensionless structure of Eq. (14) is:

16 $$\left. \begin{aligned} & C_{{fr_{x} }} = \sqrt {Re_{x} } C_{fx} = \left( {1 - \phi_{1} } \right)^{ - 2.5} \left( {1 - \phi_{2} } \right)^{ - 2.5} \sqrt {2\left( {n + 1} \right)} \;f^{\prime\prime}\left( 0 \right), \\ & C_{{fr_{z} }} = \sqrt {Re_{x} } C_{fz} = \left( {1 - \phi_{1} } \right)^{ - 2.5} \left( {1 - \phi_{2} } \right)^{ - 2.5} \sqrt {2\left( {n + 1} \right)} \;g^{\prime}\left( 0 \right), \\ & Nu_{r} = \frac{Nu}{{\sqrt {Re_{x} } }} = - \frac{{k_{hnf} }}{{k_{f} }}\sqrt {\frac{n + 1}{2}} \theta^{\prime}(0),\;Sh_{r} = \frac{Sh}{{\sqrt {Re_{x} } }} = - \sqrt {\frac{n + 1}{2}} \varphi^{\prime}(0). \\ \end{aligned} \right\}$$

## Numerical methodology

The system of Eqs. (8)–(12) are simplified to 1st order set of ODEs and solved through bvp4c package as^42,43^

17 $$\left. \begin{aligned} & \xi_{1} = f(\eta ),\;\xi_{3} = f^{\prime\prime}(\eta ),\;\xi_{5} = g^{\prime}(\eta ),\;\xi_{7} = \theta^{\prime}(\eta ),\;\xi_{9} = \varphi^{\prime}(\eta ), \\ & \xi_{2} = f^{\prime}(\eta ),\;\xi_{4} = g(\eta ),\;\xi_{6} (\eta ) = \theta (\eta ),\;\xi_{8} = \varphi (\eta ). \\ \end{aligned} \right\}$$

By putting (17) in (8–12), we get:

18 $$\xi^{\prime}_{3} + \frac{{\vartheta_{1} }}{{\vartheta_{2} }}\left( {\left( {\xi_{1} \xi_{3} - \frac{2n}{{n + 1}}Fr\xi_{2}^{2} } \right) - \frac{{\vartheta_{3} }}{{\vartheta_{1} }}\left( {\frac{2M}{{\left( {n + 1} \right)\left( {1 + m^{2} } \right)}}} \right)\xi_{2} + \lambda m\xi_{4} } \right) = 0,$$

19 $$\xi^{\prime}_{5} + \frac{{\vartheta_{1} }}{{\vartheta_{2} }}\left( {\left( {\xi_{1} \xi_{5} - \frac{2n}{{n + 1}}Fr\xi_{4} \xi_{2} } \right) - \frac{{\vartheta_{3} }}{{\vartheta_{1} }}\left( {\frac{2M}{{\left( {n + 1} \right)\left( {1 + m^{2} } \right)}}} \right)m\xi_{2} - \lambda \xi_{4} } \right) = 0,$$

20 $$\xi_{7} + Pr\,\frac{{\vartheta_{4} }}{{\vartheta_{5} }}\left( {\xi_{1} \xi_{7} + Nb\,\xi_{9} \xi_{7} + Nt\,\xi_{7}^{2} } \right) + Q_{1} \xi_{6} = 0,$$

21 $$\xi^{\prime}_{9} + \frac{Nt}{{Nb}}\xi^{\prime}_{7} + Le\xi_{1} \xi_{9} - Kr^{2} \xi_{8} = 0.$$

the transform conditions are:

22 $$\left. \begin{aligned} & \xi_{1} \left( \eta \right) = \eta \left( {\frac{1 - n}{{1 + n}}} \right),\;Nb\;\xi_{9} \left( \eta \right) + Nt\;\xi_{7} \left( \eta \right) = 0,\;\xi_{2} \left( \eta \right) = 1,\;\xi_{4} \left( \eta \right) = 0,\;\xi_{6} = 1 \\ & \xi_{2} \to 0,\;\xi_{4} \to 0,\;\xi_{6} \to 0,\;\xi_{8} \to 0\;as\;\eta \to \infty \\ \end{aligned} \right\}$$

## Result and discussion

This segment estimates the exhibition of velocity, energy and concentration outlines versus interest constraints and explain the physics behind each table and figures. The dimensionless set of ODEs (Eqs. (18)–(22)) are solved through bvp4c package.

### Velocity curve $f^{\prime}\left( \eta \right)$

**Figure 2:**
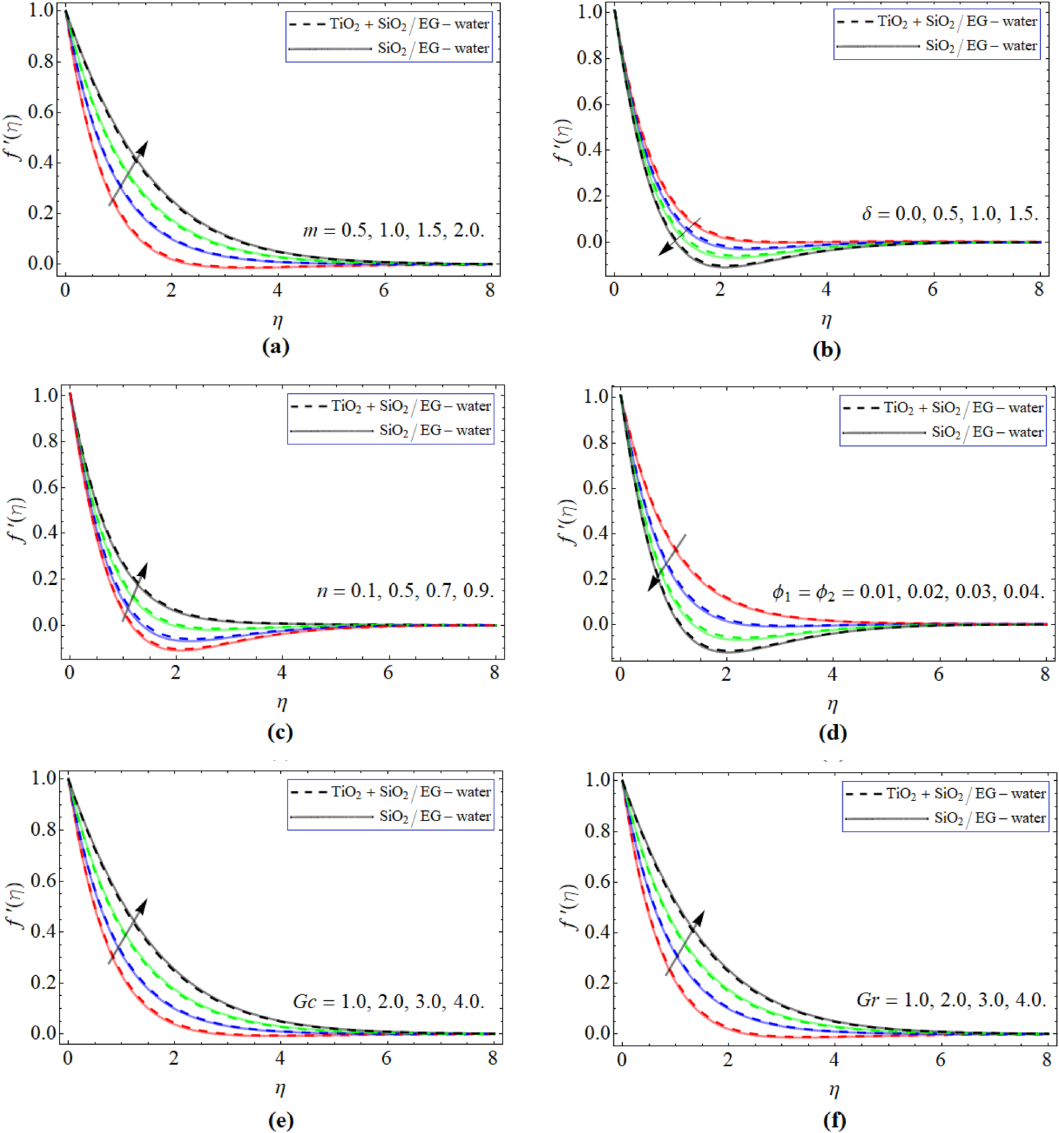
The exposition of velocity $f^{\prime}\left( \eta \right)$ curve versus constraints *m*, $\delta$, *n*, $\phi_{1} ,\,\,\phi_{2}$, G*c* and *Gr* respectively.

Figure 2a–d communicates the demonstration of velocity $f^{\prime}\left( \eta \right)$ curve versus *m*, $\delta$, *n*, $\phi_{1} ,\,\,\phi_{1}$, *Gc* and *Gr* respectively. Figure 2a,b revealed that flow velocity amplifies with the outcome of *m* and diminishes with the impact of $\delta$. Figure 2c,d exhibits that flow velocity augments with the influence of *n* and lessen with the impact of $\phi_{1} ,\,\,\phi_{2}$ respectively. The result of *n* decreases the shear stress of surface, as a result the fluid velocity improves with action of *n*. The developing amount of TiO_2_ + SiO_2_ nps grows the fluid viscosity, which triggers the retardation effect. Figure 2e,f emphasized that velocity outline boosts with the increment of thermal and mass Grashop number. The extending velocity of surface drops with the upshot of *Gc* and *Gr* which triggers the rises in the velocity outline.

**Figure 3:**
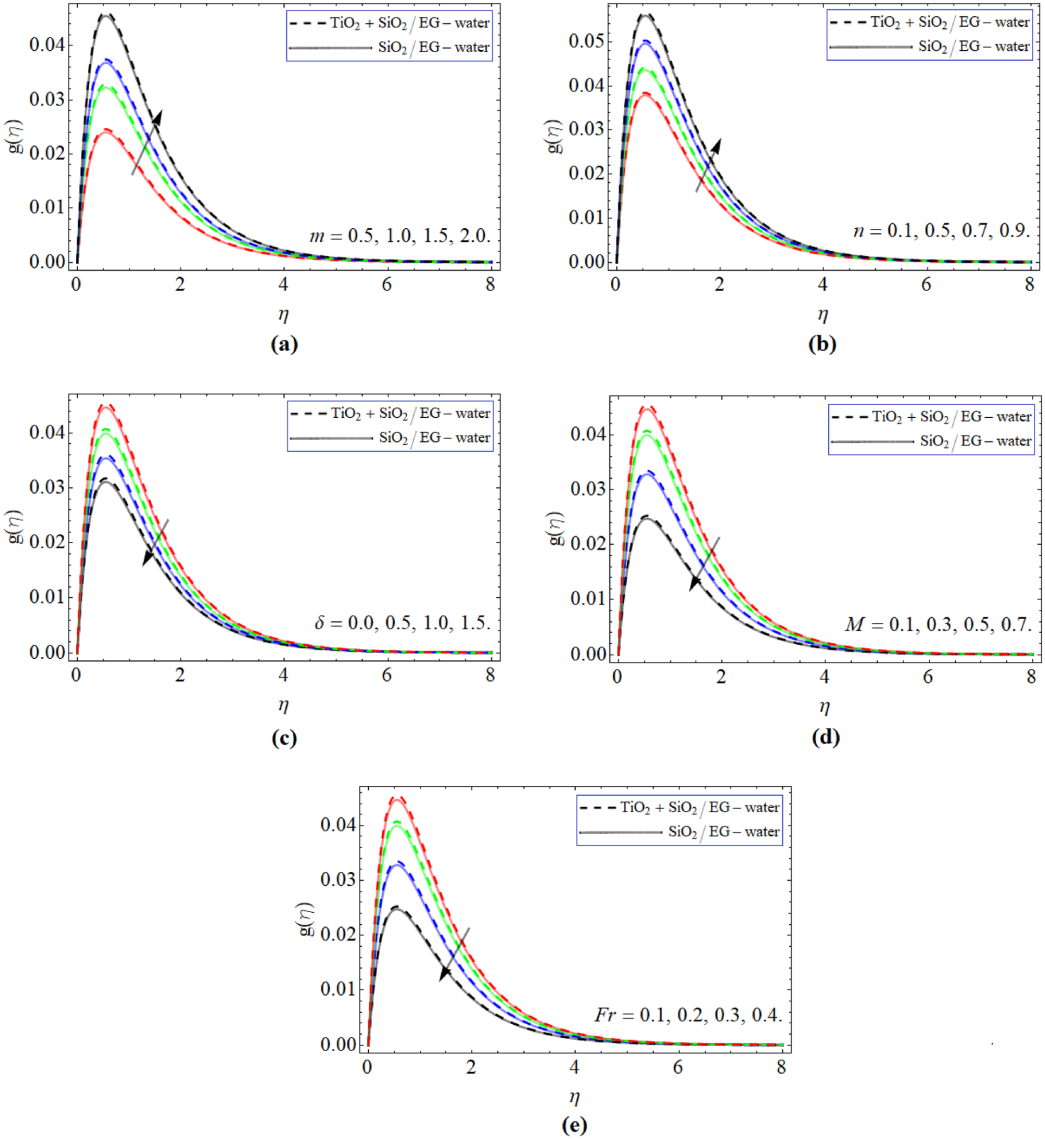
The exposition of velocity $g\left( \eta \right)$ curve versus the *m*, *n*, *M,* $\delta$ and *Fr* respectively.

Figure 3a–d demonstrated the comportment of $g\left( \eta \right)$ outline verssu parameter *m*, *n*, $\delta$, *Fr* and *M*. Figure 3a–b revealed that the flow velocity considerably upsurges with the change of *m* and *n*. While declines with the addition of $\delta$ and *M.* The magnetic upshot causes Lorentz effect, which prevents the flow moment, so the velocity profile drops. Figure 3e presents that the consequences of *Fr* pointedly de accelerates the velocity field in the radial direction.

### Energy curve $\theta \left( \eta \right)$

**Figure 4:**
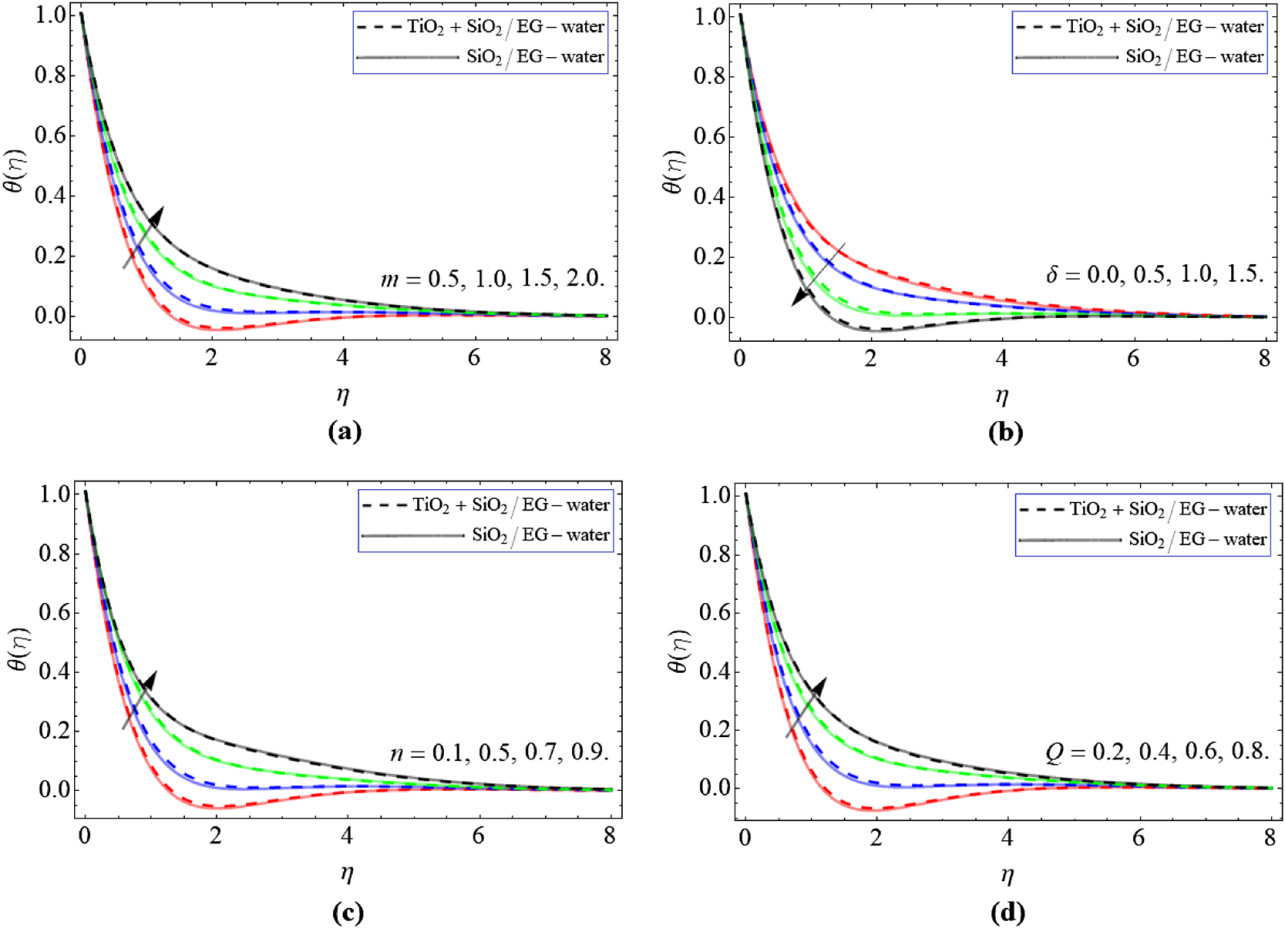
The exposition of energy $\theta \left( \eta \right)$ curve versus the *m*, $\delta$, *n* and *Q* respectively.

Figure 4a–d demonstrates the arrangement of temperature $\theta \left( \eta \right)$ curve against $\delta$, *m*, *n* and *Q*. Figure 4a,b describes that the energy $\theta \left( \eta \right)$ outline enlarged with the action of *m* and reduces under the upshot of $\delta$. Hall current result also creates confrontation, which uplifts the energy contour as perceived in Fig. 4a. Figure 4c,d represent the significances of *n* and *Q*, that their effects augment the energy profile of SiO_42_ + TiO_2_/C_2_H_6_O_2_–H_2_O hybrid nanoliquid. The consequence of *Q* term operational as a energy mediator for the nanoliquid, which directly effects the temperature outline $\theta \left( \eta \right)$.

**Figure 5:**
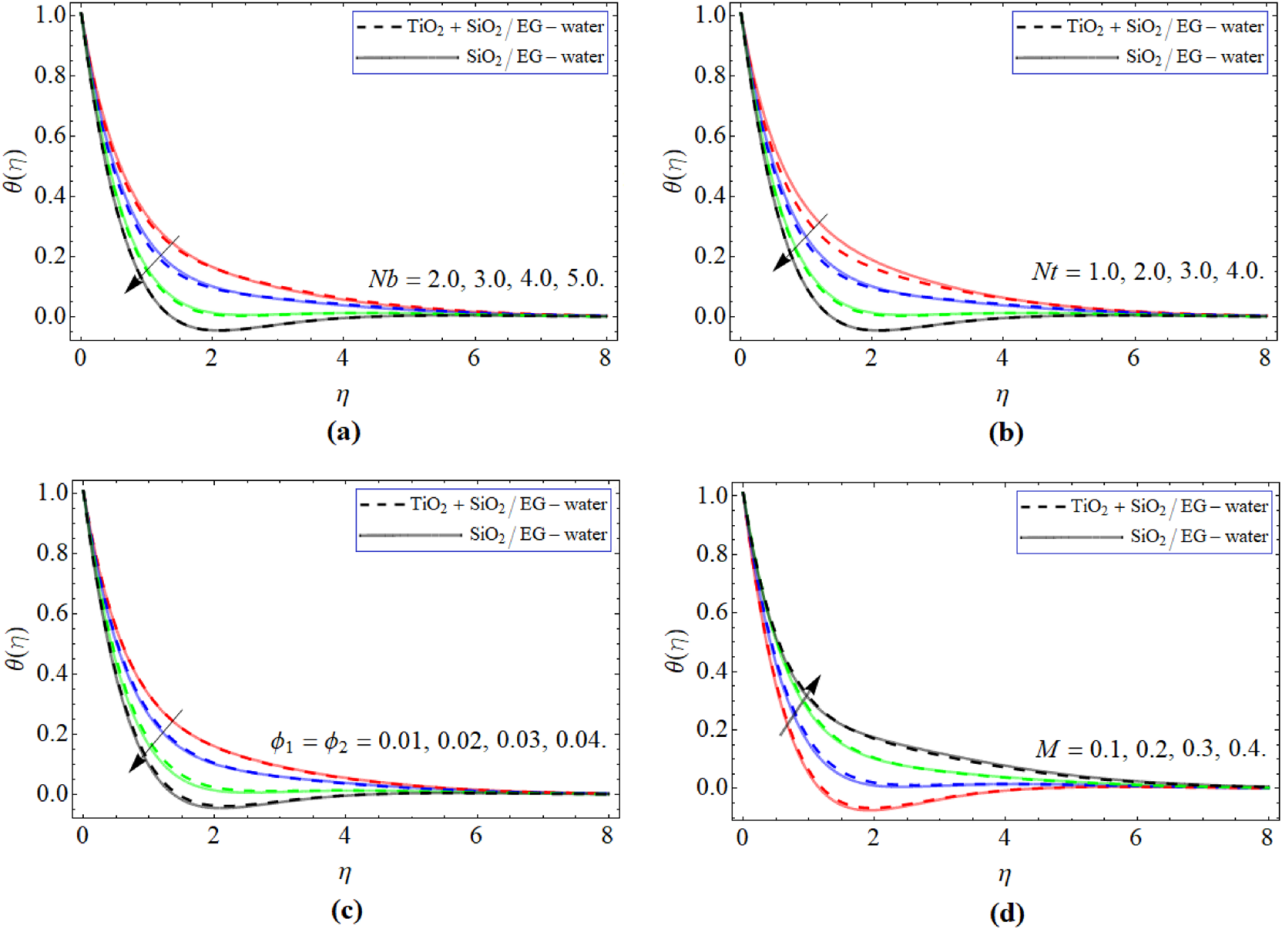
The exposition of energy $\theta \left( \eta \right)$ curve verus the *Nb*, *Nt*, $\phi_{1} ,\,\,\phi_{2}$ and *M* respectively.

Figure 5a–d emphasized the appearance of heat $\theta \left( \eta \right)$ contour relative to *Nb*, *Nt*, $\phi_{1} ,\,\,\phi_{1}$ and *M*. Figure 5a–c designated that fluid energy curve drops with the effect of *Nb*, *Nt* and $\phi_{1} ,\,\,\phi_{1}$, while enhances with the influence of magnetic field. The mounting number of nano particulates intensifies the flow velocity as well as the heat capacity of the ordinary fluid, which fallouts such scenario. As earlier deliberated that the repellant strength created by the magnetic field, absolutely effects the energy curve $\theta \left( \eta \right)$.

### Mass profile $\varphi \left( \eta \right)$

**Figure 6:**
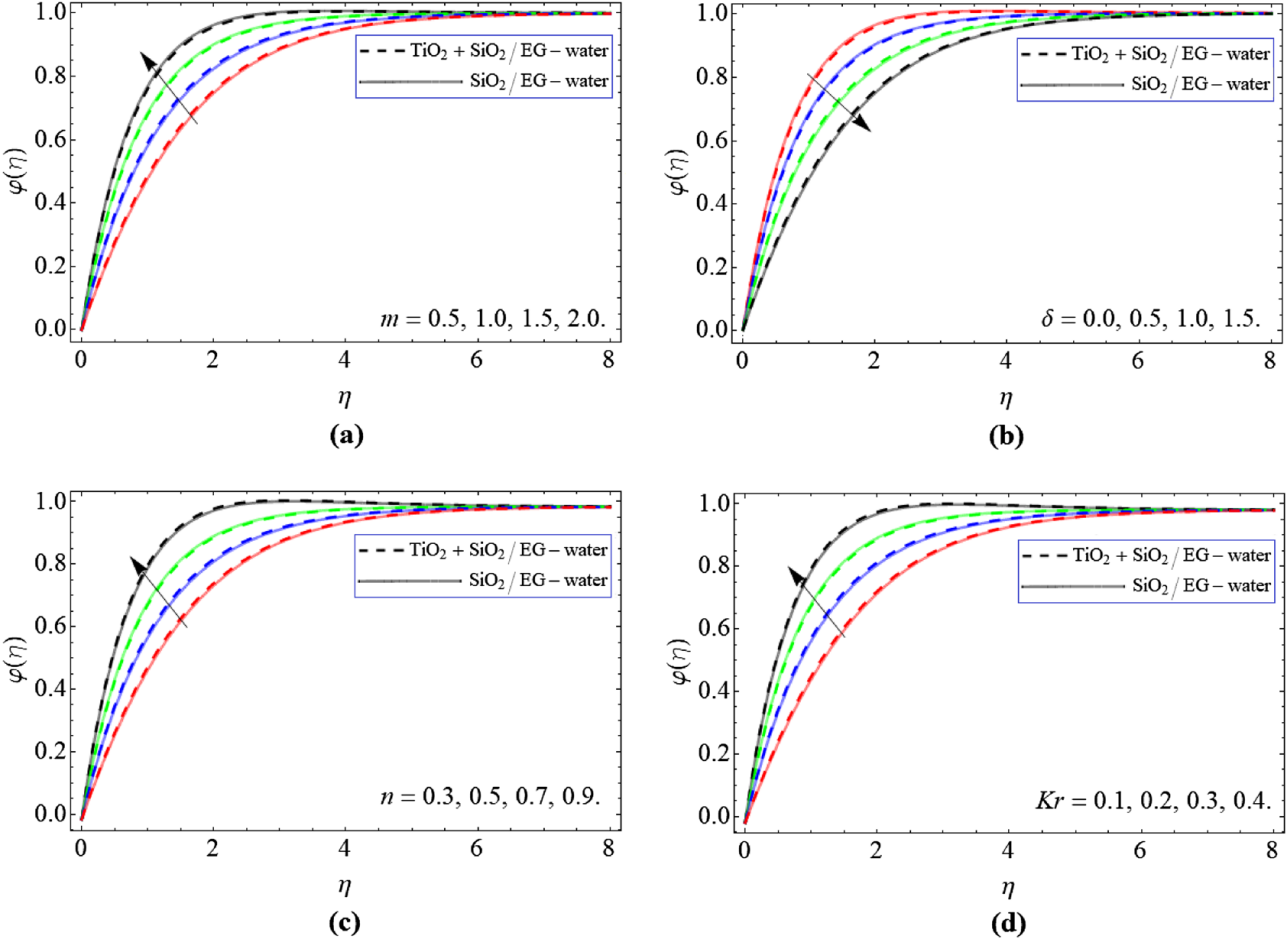
The concentration $\varphi \left( \eta \right)$ outline versus *m*, $\delta$, *n* and *Kr* respectively.

Figure 6a–c defined the exhibition of concentration $\varphi \left( \eta \right)$ contur versus *m*, $\delta$, *n* and *Kr*. The concentration conversion of hybrid nanoliquid intensify with the upshot of *m* and declines with the impact of $\delta$ as exhibited in Fig. 6a,b. Figure 6c,d described that the upshot of *Kr* and *n* both augments the mass transport. The factor *Kr* boosts the kinetic force within the nanofluid, which results in the quick communication of concentration $\varphi \left( \eta \right)$.

**Figure 7:**
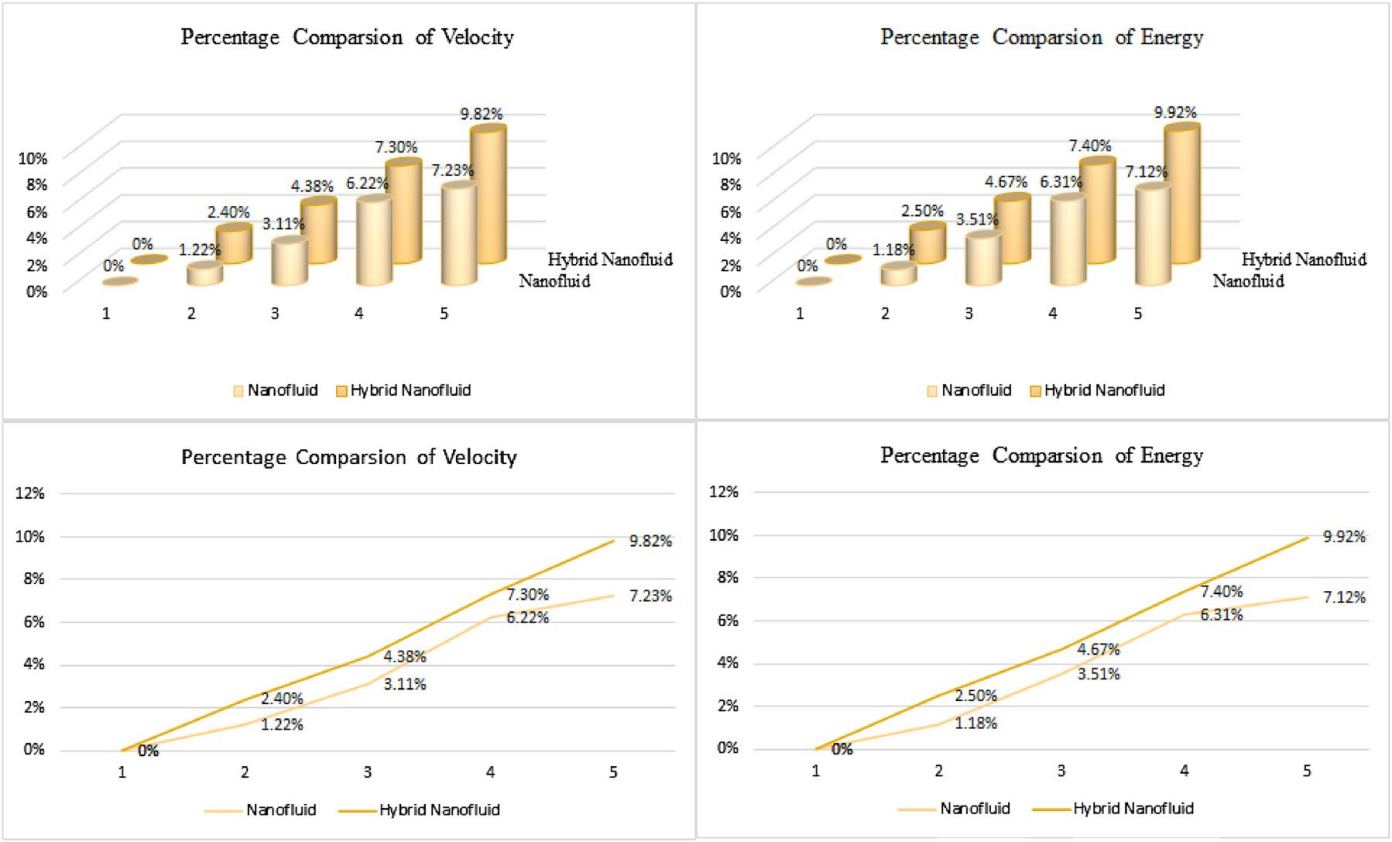
The comparation between nanoliquid and hybrid nanoliquid.

Figure 7 emphasized the relative examination of nanofluid (SiO_2_ + TiO_2_) and hybrid nanoliquid (SiO_2_ + TiO_2_/C_2_H_6_O_2_–H_2_O) for the energy and the velocity outline. Tables 1 and 2 represent the tentative values and mathematical model for SiO_2_, TiO_2_ and base fluid. Table 3 described the numerical calculation of the present outcomes with the ND solver approach, to approve the authenticity of the results. Table 4 discovered the arithmetic valuations of SiO_2_ + TiO_2_/C_2_H_6_O_2_–H_2_O hybrid nanoliquid for $C_{{f_{x} }} ,\,\,C_{{f_{z} }}$, $Nu_{r}$ and $Sh_{r}$. It is identified that the upshot of *m* augments the energy interaction rate and drag force.

**Table 1:** The tentative values of TiO_2_, TiO_2_ and C_2_H_6_O_2_–H_2_O^44^.

Nano particulates and base fluid	$\rho \;(kg/m^{3} )$	$k\;(W{/}mK)$	$Cp\;(j{/}kg\;K)$	$\sigma \;(S{/}m)$
C_2_H_6_O_2_–H_2_O	1063.8	0.387	3630	0.00509
SiO_2_	2270	1.4013	3630	$3.5 \times 10^{6}$
TiO_2_	4250	8.953	686.2	$2.38 \times 10^{6}$

**Table 2:** The mathematical model of the hybrid nanoliquid $\left( {\phi_{1} = \phi_{{SiO_{2} }} ,\,\,\,\phi_{2} = \phi_{{TiO_{2} }} } \right)$^44^.

Models
$$\frac{{\mu_{hnf} }}{{\mu_{bf} }} = \frac{1}{{\left( {1 - \phi_{{SiO_{2} }} - \phi_{{TiO_{2} }} } \right)^{2} }}$$
$$\frac{{\rho_{hnf} }}{{\rho_{bf} }} = \phi_{{SiO_{2} }} \left( {\frac{{\rho_{{SiO_{2} }} }}{{\rho_{bf} }}} \right) + \phi_{{TiO_{2} }} \left( {\frac{{\rho_{{TiO_{2} }} }}{{\rho_{bf} }}} \right) + \left( {1 - \phi_{{SiO_{2} }} - \phi_{{TiO_{2} }} } \right)$$
$$\frac{{(\rho C_{p} )_{hnf} }}{{(\rho C_{p} )_{bf} }} = \phi_{{SiO_{2} }} \left( {\frac{{(\rho C_{p} )_{{SiO_{2} }} }}{{(\rho C_{p} )_{bf} }}} \right) + \phi_{{TiO_{2} }} \left( {\frac{{(\rho C_{p} )_{{TiO_{2} }} }}{{(\rho C_{p} )_{bf} }}} \right) + \left( {1 - \phi_{{SiO_{2} }} - \phi_{{TiO_{2} }} } \right)$$
$$\frac{{(\rho \beta_{T} )_{hnf} }}{{(\rho \beta_{T} )_{bf} }} = \phi_{{SiO_{2} }} \left( {\frac{{(\rho \beta_{T} )_{{SiO_{2} }} }}{{(\rho \beta_{T} )_{bf} }}} \right) + \phi_{{TiO_{2} }} \left( {\frac{{(\rho \beta_{T} )_{{TiO_{2} }} }}{{(\rho \beta_{T} )_{bf} }}} \right) + \left( {1 - \phi_{{SiO_{2} }} - \phi_{{TiO_{2} }} } \right)$$
$$\frac{{k_{hnf} }}{{k_{bf} }} = \left[ {\frac{{\left( {\frac{{\phi_{{SiO_{2} }} k_{{SiO_{2} }} + \phi_{{TiO_{2} }} k_{{TiO_{2} }} }}{{\phi_{{SiO_{2} }} + \phi_{{TiO_{2} }} }}} \right) + 2k_{bf} + 2\left( {\phi_{{SiO_{2} }} k_{{SiO_{2} }} + \phi_{{TiO_{2} }} k_{{TiO_{2} }} } \right) - 2\left( {\phi_{{SiO_{2} }} + \phi_{{TiO_{2} }} } \right)k_{bf} }}{{\left( {\frac{{\phi_{{SiO_{2} }} k_{{SiO_{2} }} + \phi_{{TiO_{2} }} k_{{TiO_{2} }} }}{{\phi_{{SiO_{2} }} + \phi_{{TiO_{2} }} }}} \right) + 2k_{bf} - 2\left( {k_{{SiO_{2} }} \phi_{{SiO_{2} }} + k_{{TiO_{2} }} \phi_{{TiO_{2} }} } \right) + 2\left( {\phi_{{SiO_{2} }} + \phi_{{TiO_{2} }} } \right)k_{bf} }}} \right]$$
$$\frac{{\sigma_{hnf} }}{{\sigma_{bf} }} = \left[ {\frac{{\left( {\frac{{\sigma_{{SiO_{2} }} \phi_{{SiO_{2} }} + \phi_{{TiO_{2} }} \sigma_{{TiO_{2} }} }}{{\phi_{{TiO_{2} }} + \phi_{{SiO_{2} }} }}} \right) + 2\sigma_{bf} + 2\left( {\sigma_{{SiO_{2} }} \phi_{{SiO_{2} }} + \phi_{{TiO_{2} }} \sigma_{{TiO_{2} }} } \right) - 2\left( {\phi_{{SiO_{2} }} + \phi_{{TiO_{2} }} } \right)\sigma_{bf} }}{{\left( {\frac{{\phi_{{SiO_{2} }} \sigma_{{SiO_{2} }} + \phi_{{TiO_{2} }} \sigma_{{TiO_{2} }} }}{{\phi_{{SiO_{2} }} + \phi_{{TiO_{2} }} }}} \right) + 2\sigma_{bf} - \left( {\sigma_{{SiO_{2} }} \phi_{{SiO_{2} }} + \phi_{{TiO_{2} }} \sigma_{{TiO_{2} }} } \right) + \left( {\phi_{{SiO_{2} }} + \phi_{{TiO_{2} }} } \right)\sigma_{bf} }}} \right]$$

**Table 3:** The numerical outcomes for skin friction $- f^{\prime\prime}(0)$.

*n*	Ref.^41^ $- f^{\prime\prime}(0)$	Ref.^45^ $- f^{\prime\prime}(0)$	bvp4c	ND-solve
			Current outcomes $- f^{\prime\prime}(0)$	Current outcomes $- f^{\prime\prime}(0)$
1.0	1.000001	1.0000	1.000000	1.000000
2.0	1.023410	1.0234	1.023511	1.023500
3.0	1.035871	1.0359	1.035970	1.035961
5.0	1.048615	1.0486	1.048514	1.048503
7.0	1.055049	1.0550	1.055248	1.055227
9.0	1.058920	1.0589	1.058941	1.058901
10	1.060329	1.0603	1.060428	1.060407

**Table 4:** The numerical results for $\left( {C_{{f_{x} }} ,\,\,C_{{f_{z} }} } \right)$,$Nu_{r}$ and $Sh_{r}$.

*m*	$\delta$	*N*	$C_{{f_{x} }}$	$C_{{f_{z} }}$	$Nu_{r}$	$Sh_{r}$
0.2			− 1.689775	0.418367	1.225167	3.371834
0.4			− 1.286627	0.685572	1.259011	3.234460
0.6			− 1.865864	0.726487	1.289550	3.104671
0.8			− 0.1646348	0.765092	1.412453	3.006296
1.0			− 1.496956	0.791047	1.429020	2.935582
	0.1		− 1.509798	0.420456	1.18262	1.723911
	0.2		− 1.689775	0.418367	1.625167	2.371834
	0.3		− 1.877942	0.414772	1.701782	6.058711
	0.4		− 1.074212	0.409747	2.303375	8.311254
	0.5		− 1.278457	0.403394	2.723852	10.47904
		0.2	− 1.689775	0.418367	1.325167	5.371834
		0.3	− 2.214652	0.497395	1.253300	3.381260
		0.4	− 2.686005	0.567704	1.265827	2.474134
		0.5	− 3.116150	0.631884	1.222736	0.677470
		0.6	− 3.513876	0.691371	1.206127	0.572587

## Conclusion

We have examined the flow features of hybrid nanoliquid through a slender stretching surface. The consequences of second order exothermic reaction, heat source, Hall current and magnetic fields are all also described. The modeled equations are assessed by using the numerical approach bvp4c package. The important conclusions are:

- The $f^{\prime}\left( \eta \right)$ outline augments with the outcome of *n* and *m* and while reduces with the rising quanitity of nano particulates $\phi_{1} ,\,\,\phi_{2}$ and parameter $\delta .$
- The velocity $g\left( \eta \right)$ curve substantially upsurges with effect of *n* and *m*. While decresaes with the effect of $\delta$ and *M.*
- The energy $\theta \left( \eta \right)$ curve is enhances with the variation of *m* and diminishes with the $\delta$.
- The energy $\theta \left( \eta \right)$ contour diminish with the influence of *Nb*, *Nt* and $\phi_{1} ,\,\,\phi_{1}$, while boosts with the outcome of magnetic effect.
- The concentration $\varphi \left( \eta \right)$ outline of hybrid nanoliquid improves with the upshot of *Kr* and *n.*
- The current model may be expanded to other type of fluid and can be used different chemical composition nanoparticles in the base fluid for desire output. Furthermore, different numerical, analytical and fractional methods can also be used to solve such problems.

## Data Availability

All data used in this manuscript have been presented within the article.

## Abbreviations

*n*: Power index
*U*_*0*_: Reference velocity
$$m$$: Hall current
*K*^***^: Permeability factor
2D: Two-dimension
$F$: Non-uniform inertia factor
*T*: Temperature
*Nt*: Thermodiffusion
*Pr*: Prandtl number
$$\mu_{hnf}$$: Viscosity
$$(\rho C_{p} )_{hnf}$$: Thermal capacity
$$k_{hnf}$$: Thermal conductivity
$\phi_{1} = \phi_{{SiO_{2} }}$: Nanoparticles volume friction
C_2_H_6_O_2_: Ethyne glycol
$$\left( {u,\,v,\,w} \right)$$: Velocity component
*A*: Stretching constant
$$U_{w}$$: Stretching velocity
*Kc*^*2*^: Chemical reaction rate
*Q*_*0*_: Heat source
MHD: Magnetohydrodynamics
*C*: Concentration
$$\lambda$$: Porosity factor
*Nb*: Brownian motion
*Gr*: Grashof number
$$\rho_{hnf}$$: Density
$$(\rho \beta_{T} )_{hnf}$$: Thermal expansion
$$\sigma_{hnf}$$: Electrical conductivity
$\phi_{2} = \phi_{{TiO_{2} }}$: Nanoparticles volume friction
TiO_2_: Titanium dioxide
SiO_2_: Silicon dioxide

## References

[1] Mjankwi, M. A., Masanja, V. G., Mureithi, E. W., & James, M. N. O. (2019). Unsteady MHD flow of nanofluid with variable properties over a stretching sheet in the presence of thermal radiation and chemical reaction. *Int. J. Math. Math. Sci*. (2019).

[2] Varun Kumar RS, Punith Gowda RJ, Naveen Kumar R, Radhika M, Prasannakumara BC (2021). Two-phase flow of dusty fluid with suspended hybrid nanoparticles over a stretching cylinder with modified Fourier heat flux. SN Appl. Sci..

[3] Varun Kumar RS, Alhadhrami A, Punith Gowda RJ, Naveen Kumar R, Prasannakumara BC (2021). Exploration of Arrhenius activation energy on hybrid nanofluid flow over a curved stretchable surface. ZAMM-J. Appl. Math. Mech./Zeitschrift für Angewandte Mathematik und Mechanik.

[4] Gul T, Khan A, Bilal M, Alreshidi NA, Mukhtar S, Shah Z, Kumam P (2020). Magnetic dipole impact on the hybrid nanofluid flow over an extending surface. Sci. Rep..

[5] Bilal M, Saeed A, Selim MM, Gul T, Ali I, Kumam P (2021). Comparative numerical analysis of Maxwell's time-dependent thermo-diffusive flow through a stretching cylinder. Case Stud. Thermal Eng..

[6] SafwaKhashiie N, MdArifin N, Hafidzuddin EH, Wahi N (2019). Dual stratified nanofluid flow past a permeable shrinking/stretching sheet using a non-Fourier energy model. Appl. Sci..

[7] Hussain A, Arshad M, Rehman A, Hassan A, Elagan SK, Ahmad H, Ishan A (2021). Three-dimensional water-based magneto-hydrodynamic rotating nanofluid flow over a linear extending sheet and heat transport analysis: A numerical approach. Energies.

[8] Shuaib M, Bilal M, Khan MA, Malebary SJ (2020). Fractional analysis of viscous fluid flow with heat and mass transfer over a flexible rotating disk. Comput. Model. Eng. Sci..

[9] Hussain A, Rehman A, Ahmed N, El-Shafay AS, Najati SA, Almaliki AH, Sherif ESM (2022). Heat transfer and flow characteristics of pseudoplastic nanomaterial liquid flowing over the slender cylinder with variable characteristics. Curr. Comput.-Aided Drug Des..

[10] Uddin Z, Vishwak KS, Harmand S (2021). Numerical duality of MHD stagnation point flow and heat transfer of nanofluid past a shrinking/stretching sheet: Metaheuristic approach. Chin. J. Phys..

[11] Rasool G, Shafiq A, Alqarni MS, Wakif A, Khan I, Bhutta MS (2021). Numerical scrutinization of Darcy–Forchheimer relation in convective magnetohydrodynamic nanofluid flow bounded by nonlinear stretching surface in the perspective of heat and mass transfer. Micromachines.

[12] Ahmad S, Khan MN, Nadeem S, Rehman A, Ahmad H, Ali R (2021). Impact of Joule heating and multiple slips on a Maxwell nanofluid flow past a slendering surface. Commun. Theor. Phys..

[13] Ahmadian A, Bilal M, Khan MA, Asjad MI (2020). The non-Newtonian maxwell nanofluid flow between two parallel rotating disks under the effects of magnetic field. Sci. Rep..

[14] Song YQ, Khan MI, Qayyum S, Gowda RP, Kumar RN, Prasannakumara BC (2021). Physical impact of thermo-diffusion and diffusion-thermo on Marangoni convective flow of hybrid nanofluid (MnZiFe_2_O_4_–NiZnFe_2_O_4_–H_2_O) with nonlinear heat source/sink and radiative heat flux. Mod. Phys. Lett. B.

[15] Khan MI, Qayyum S, Shah F, Kumar RN, Gowda RP, Prasannakumara BC (2021). Marangoni convective flow of hybrid nanofluid (MnZnFe_2_O_4_–NiZnFe_2_O_4_–H_2_O) with Darcy Forchheimer medium. Ain Shams Eng. J..

[16] Li YX, Khan MI, Gowda RP, Ali A, Farooq S, Chu YM, Khan SU (2021). Dynamics of aluminum oxide and copper hybrid nanofluid in nonlinear mixed Marangoni convective flow with entropy generation: Applications to renewable energy. Chin. J. Phys..

[17] Kazemi M, Ghobadi M, Mirzaie A (2018). Cobalt ferrite nanoparticles (CoFe_2_O_4_ MNPs) as catalyst and support: magnetically recoverable nanocatalysts in organic synthesis. Nanotechnol. Rev..

[18] Traciak J, Sobczak J, Kuzioła R, Wasąg J, Żyła G (2022). Surface and optical properties of ethylene glycol-based nanofluids containing silicon dioxide nanoparticles: An experimental study. J. Therm. Anal. Calorim..

[19] Bhatti MM, Öztop HF, Ellahi R, Sarris IE, Doranehgard MH (2022). Insight into the investigation of diamond (C) and Silica (SiO_2_) nanoparticles suspended in water-based hybrid nanofluid with application in solar collector. J. Mol. Liq..

[20] Ahmed J, Shahzad A, Farooq A, Kamran M, Ud-Din Khan S, Ud-Din Khan S (2021). Thermal analysis in swirling flow of titanium dioxide–aluminum oxide water hybrid nanofluid over a rotating cylinder. J. Therm. Anal. Calorim..

[21] Khashi’ie NS, Md Arifin N, Pop I, Nazar R (2022). Melting heat transfer in hybrid nanofluid flow along a moving surface. J. Thermal Anal. Calorim..

[22] Khashi'ie, N. S., Waini, I., Zokri, S. M., Kasim, A. R. M., Arifin, N. M., & Pop, I. (2021). Stagnation point flow of a second-grade hybrid nanofluid induced by a Riga plate. *Int. J. Numer. Methods Heat Fluid Flow*.

[23] Alwawi, F., Sulaiman, I. M., Swalmeh, M. Z., & Yaseen, N. (2022). Energy transport boosters of magneto micropolar fluid flowing past a cylinder: A case of laminar combined convection. in *Proceedings of the Institution of Mechanical Engineers, Part C: Journal of Mechanical Engineering Science*, 09544062221111055.

[24] Abbasi, A., Gul, M., Farooq, W., Khan, S. U., Aydi, A., Ayadi, B., *et al*. (2022). A comparative thermal investigation for modified hybrid nanofluid model (Al_2_O_3_–SiO_2_–TiO_2_)/(C2H_6_O_2_) due curved radiated surface. *Case Stud. Thermal Eng*. 102295.

[25] Khashi’ie NS, Waini I, Ishak A, Pop I (2022). Blasius flow over a permeable moving flat plate containing Cu–Al_2_O_3_ hybrid nanoparticles with viscous dissipation and radiative heat transfer. Mathematics.

[26] De P (2019). Soret-Dufour effects on unsteady flow of convective Eyring-Powell magneto nanofluids over a semi-infinite vertical plate. BioNanoScience.

[27] Mondal, H., De, P., Goqo, S., & Sibanda, P. (2020). A numerical study of nanofluid flow over a porous vertical plate with internal heat generation and nonlinear thermal radiation. *J. Porous Media*. **23**(6).

[28] Khan U, Ahmed N, Mohyud-Din ST, Alsulami MD, Khan I (2022). A novel analysis of heat transfer in the nanofluid composed by nanodimaond and silver nanomaterials: numerical investigation. Sci. Rep..

[29] Xiong PY, Khan MI, Gowda RP, Kumar RN, Prasannakumara BC, Chu YM (2021). Comparative analysis of (Zinc ferrite, Nickel Zinc ferrite) hybrid nanofluids slip flow with entropy generation. Mod. Phys. Lett. B.

[30] Kumar RN, Gowda RP, Abusorrah AM, Mahrous YM, Abu-Hamdeh NH, Issakhov A (2021). Impact of magnetic dipole on ferromagnetic hybrid nanofluid flow over a stretching cylinder. Phys. Scr..

[31] Hamid A, Naveen Kumar R, Punith Gowda RJ, Varun Kumar RS, Khan SU, Ijaz Khan M (2021). Impact of Hall current and homogenous–heterogenous reactions on MHD flow of GO-MoS2/water (H2O)-ethylene glycol (C2H6O2) hybrid nanofluid past a vertical stretching surface. Waves Random Complex Media.

[32] Punith Gowda RJ, Naveen Kumar R, Jyothi AM, Prasannakumara BC, Sarris IE (2021). Impact of binary chemical reaction and activation energy on heat and mass transfer of marangoni driven boundary layer flow of a non-Newtonian nanofluid. Processes.

[33] De P, Gorji MR (2020). Activation energy and binary chemical reaction on unsteady MHD Williamson nanofluid containing motile gyrotactic micro-organisms. Heat Transfer.

[34] Sangeetha, E., & De, P. (2021). Bioconvection in nanofluid flow embedded in non-Darcy porous medium with viscous dissipation and Ohmic heating. *J. Porous Media*. **24**(1).

[35] Khan MN, Nadeem S (2021). A comparative study between linear and exponential stretching sheet with double stratification of a rotating Maxwell nanofluid flow. Surfaces Interfaces.

[36] Acharya N, Mabood F, Shahzad SA, Badruddin IA (2022). Hydrothermal variations of radiative nanofluid flow by the influence of nanoparticles diameter and nanolayer. Int. Commun. Heat Mass Transfer.

[37] Khashi'ie NS, Arifin NM, Pop I (2022). Magnetohydrodynamics (MHD) boundary layer flow of hybrid nanofluid over a moving plate with Joule heating. Alex. Eng. J..

[38] Waini I, Khashi’ie NS, Kasim ARM, Zainal NA, Hamzah KB, Md Arifin N, Pop I (2022). Unsteady magnetohydrodynamics (MHD) flow of hybrid ferrofluid due to a rotating disk. Mathematics.

[39] Gouran S, Mohsenian S, Ghasemi SE (2022). Theoretical analysis on MHD nanofluid flow between two concentric cylinders using efficient computational techniques. Alex. Eng. J..

[40] Shamshuddin MD, Ibrahim W (2022). Finite element numerical technique for magneto-micropolar nanofluid flow filled with chemically reactive casson fluid between parallel plates subjected to rotatory system with electrical and Hall currents. Int. J. Model. Simulat..

[41] Das K, Giri SS, Kundu PK (2021). Influence of Hall current effect on hybrid nanofluid flow over a slender stretching sheet with zero nanoparticle flux. Heat Transfer.

[42] Shuaib M, Shah RA, Durrani I, Bilal M (2020). Electrokinetic viscous rotating disk flow of Poisson-Nernst-Planck equation for ion transport. J. Mol. Liq..

[43] Shuaib M, Shah RA, Bilal M (2021). Von-Karman rotating flow in variable magnetic field with variable physical properties. Adv. Mech. Eng..

[44] Haq, I., Bilal, M., Ahammad, N. A., Ghoneim, M. E., Ali, A., & Weera, W. (2022). Mixed convection nanofluid flow with heat source and chemical reaction over an inclined irregular surface. *ACS Omega*. 10.1021/acsomega.2c03919 PMC9435030 36061645

[45] Khader MM, Megahed AM (2015). Boundary layer flow due to a stretching sheet with a variable thickness and slip velocity. J. Appl. Mech. Tech. Phys..

